# Shallominthe Active Antimicrobial Constituent of Persian Shallot in Treatment of Oral Herpes: A Double-Blind Randomized Clinical Trial

**DOI:** 10.17795/jjnpp-17372

**Published:** 2014-08-01

**Authors:** Mohammad Hassan Pipelzadeh, Mansour Amin, Abolfazl Shiravi Khozani, Mohammad Radmanesh

**Affiliations:** 1 Department of Pharmacology and Toxicology Research Centre, Ahvaz Jundishapur University of Medical Sciences, Ahvaz, IR Iran; 2 Department of Microbiology, Infection and Tropical Diseases Research Center, School of Medicine, Ahvaz Jundishapur University of Medical Sciences, Ahvaz, IR Iran; 3 Department of Dermatology, Laser Research Center, Ahvaz Jundishapur University of Medical Sciences, Ahvaz, IR Iran

**Keywords:** Herpes simples, Cold Sore, Shallomin, Treatment

## Abstract

**Background:**

Previous studies showed that shallomin, the active antimicrobial constituent of Persian shallot, has a wide range of antibacterial and antifungal properties.

**Objectives:**

The objective of this randomized clinical trial was to evaluate the effectiveness of topical shallomin alcoholic solution in treatment of cold sore.

**Patients and Methods:**

A total of 60 volunteers who met the inclusion criteria were randomly allocated to two equal groups to hourly apply topical of either 0.5% shallomin alcoholic solution or placebo within the first 24 hours of developing cold sores. All the cases were reassessed at six-hour intervals.

**Results:**

The cold sores were cleared within six hours among 30% of cases who received shallomin solution and the remaining of the cases in this group were cleared between 6six to 24 hours of application. In the placebo group, clearance of the sores occurred in four cases between 48 to 72 hours and the remaining of cases were cleared after 72 hours.

**Conclusions:**

The results of this study demonstrated that shallomin is a useful natural remedy in preventing the progression and treatment of cold sores and can significantly reduce the duration of ulceration.

## 1. Background

There are two types of herpes simplex virus (HSV); type 1 (HSV-1) usually causes oral herpes while type 2 (HSV-2) causes genital herpes. HSV-1 is a contagious virus that causes oral herpes or cold sores. More serious viral infections that arise from HSV include ocular herpes and herpetic encephalitis. Oral herpes is the most common form of infection that occurs in the face, particularly the lips and mouth. At present, there is no complete cure from this recurrent and self-limited disease and prescribed medications only shorten the duration of healing and severity of the infection. Furthermore, once individuals are infected with this HSV, they become a carrier of the virus for life and experiences periodic or sporadic recurrence of infection with variable degrees of severity. On the other hand, infection with HSV-1 has been associated with cognitive deficit in bipolar disorder (1), Alzheimer disease (2), and neonatal herpes (3).

Treatment modalities for HSV vary widely and have different degrees of effectiveness. There are several antiviral drugs that are effective for treating HSV including acyclovir, valacyclovir, famciclovir, and penciclovir. In addition, certain dietary supplements and alternative medicine drugs are claimed to be beneficial, which include Echinacea, L-lysine, Zn, bee products, and aloe vera (4); however, the evidences supporting their effectiveness have not been confirmed in larger randomized controlled studies (5).

The *Allium hirtifolium* (Persian shallot) belongs to the genus *Allium*, which includes onion and garlic, and is a grow-wild plant in the Zagros Mountains, Iran. Previous in vitro studies showed that the crude extract of *A. hirtifolium* bulb has a heat-stable antimicrobial property against a variety of pathogenic bacteria including *Pseudomonas aeruginosa*, *Staphylococcus aureus*, *Listeria monocytogenes*, *Bacillus cereus*, *Serratia marcescens*, *Escherichia coli*, various* Campylobacter *species, *Salmonella typhi*, *Salmonella paratyphi* A, *Proteus mirabilis*, and *Shigella* species with a minimum inhibitory concentration (MIC) ranging from 5 to 40 μg/mL (6). In addition, the crude extract showed fungistatic and fungicidal activity against pathogenic fungi including *Microsporum gypseum*, *Aureobasidium pullulans*, *Trichophyton mentagrophyte*, *Trichophyton rubrum*, *Fusarium oxysporum*, *Saccharomyces cerevisiae*, *Aspergillus niger*, *Aspergillus* *flavus*, *Aspergillus fumigatus*, and *Candida albicans* with a MIC ranging from 0.15 to 20 μg/mL. The active compound responsible for the antimicrobial properties is a flavonoid with the general formula of C_14_H_8_O_8_, named shallomin (7). More recently, it was demonstrated that shallomin has a low toxicity profile (8) and the aqueous mouth-rinse extract produced more prolonged antibacterial activity than chlorhexidine (9).

In support of these findings, previous studies performed on other species belonging to *Allium* demonstrated significant antibacterial, antifungal, antiviral, antiprotozoal, and anthelmintic properties (10). In addition, there are ample evidences that show favorable influence of this plant on improvement of some diseases such as diabetes, arthritis, common cold, flu, stress, fever, cough, headache, hemorrhoids, asthma, arteriosclerosis, and cancer (11).

## 2. Objectives

These unique properties, which are not commonly found in current antimicrobial agents, confer that the antiviral properties of this medicinal plant might have clinical significance in treatment of cold sores. No previous studies have attempted to test this property of shallomin. The aim of the present study was to test the antiviral effectiveness of shallomin in human volunteers.

## 3. Materials and Methods

### 3.1. Extraction and Preparation of Shallomin Roll-on Sticks

**Figure 1.**
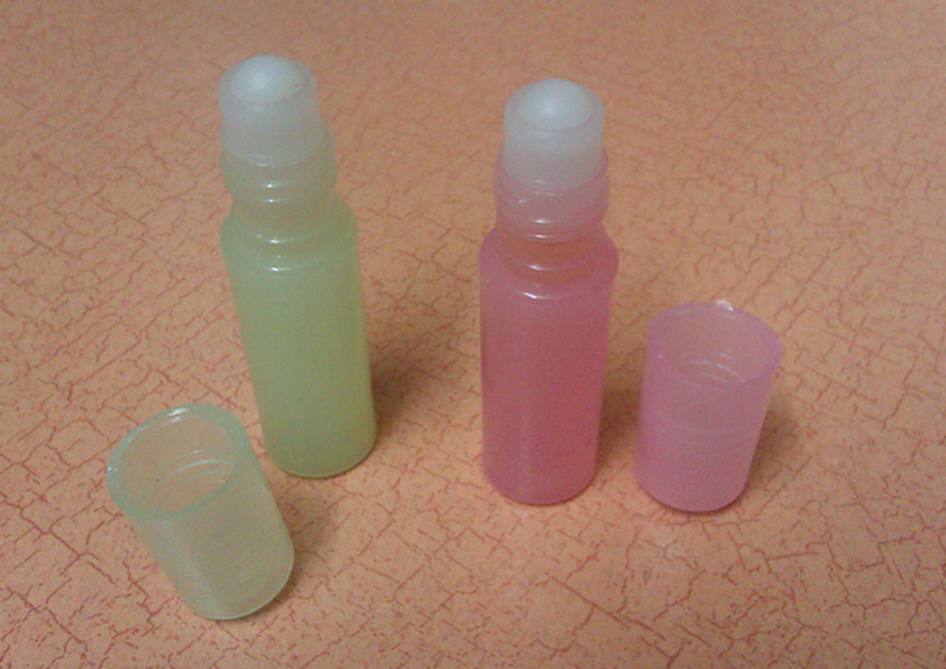
A Photographic Illustration of the Shallomin Roll-on Used in the Study

The method of extraction of shallomin was adopted as previously described (8). In short, 500 g of fresh shallot bulbs (collected from Zagros mountains, 50 km to Dezful City, south of Iran, in spring season; identified by National Chemical Laboratory, Pune, India) were washed thoroughly in water and broken down into small pieces by an electrical grinder, soaked in 500 mL of distilled water for 24 hours, and filtered by Wattman No. 1. The filtrate was mixed with 50% ethyl acetate/water mixture for a further 24 hours. The ethyl acetate fraction was separated and allowed to evaporate under a hood. The precipitate was collected and weighed, and finally, solution of 0.5% in absolute alcohol was prepared and packed in 2 mL roll-on stick containers (Figure 1) and stored in a cool room at 4℃ until used.

### 3.2. Subjects and Experimental Protocol

A total of 30 volunteers who met the inclusion criteria were recruited in each arm of intervention. Enrolled participants were male or female students of Ahvaz Jundishapur University of Medical Sciences who were diagnosed with cold sore by a dermatologist member of the research team. All the subjects had developed cold sores within 24 hours before diagnosis. The subjects in the test group were asked to apply the roll-on stick on the cold sore hourly until the tingling and the sores cleared. The control group was asked to apply vehicle only (absolute alcohol) prepared in similar looking containers hourly. The sores in both groups were assessed by a blinded dermatologist hourly during the first six hours and then at 24, 48, 72, and 84 hours after initiation of intervention or until the skin cleared. The cases were assessed during this period in microbiology or in dermatology departments of Emam Khomeini Hospital of Ahvaz Jundishapur University of Medical Sciences.

### 3.3. Statistical Assessment

The data were analyzed in SPSS 16.0 using Chi squared test with a P < 0.05 was considered significant.

### 3.4. Ethical Considerations

The ethical committee of Ahvaz Jundishapur University of Medical Sciences has approved the project protocol. All the participants signed an informed written consent before commencement of the study.

## 4. Results

**Figure 2.**
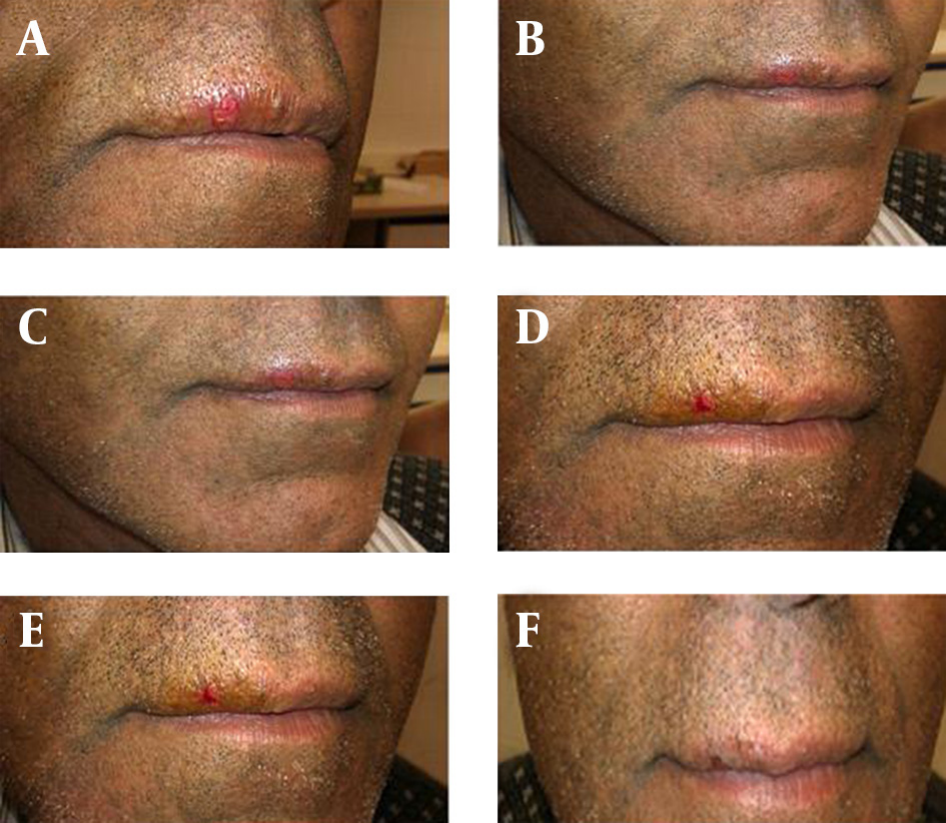
A Photographic Illustration of a Shallomin Alcoholic Solution-Treated Case Before initiation of treatment (0) and after hourly treatment (1 to 5 hours).

The tingling and cold sores cleared within six hours in nine cases (30%) of the shallomin group and within 24 hours in the remaining patients. In the placebo group, the cold sores cleared in four cases (13.3%) between 48 to 72 hours and cleared after 72 hours in the remaining cases (Table 1). A photograph of a case with cold sore before and after hourly application of shallomin up to five hours is shown in Figure 2.

**Table 1.** Duration of Treatment and Number of Cases Cleared of Cold Sores Following Treatment With 0.5% Shallomin and Placebo (n = 30)

Duration of Treatment, h	Cleared Cases, No.	
	Shallomin Group	Placebo (Absolute Alcohol) Group
**Within 6**	9^a^	-
**6-24**	21^a^	-
**24-48**	-	-
**48-72**	-	4 ^b^
**> 72**	-	26 ^b^

^a^ P < 0.001 between treatment groups; Chi-squared test.

^b^P < 0.001 between placebo- and shallomin-treated groups.

## 5. Discussion

In the present study, we examined the effectiveness of shallomin, the active antimicrobial constituent of Persian shallot, in treatment of patients who developed cold sore within the previous 24 hours. The results showed that 0.5% shallomin led to a rapid clearance of the sores after five to six hours following hourly topical application with no significant side effects and with a minimal crust or scab formation.

Previous studies have shown that different modalities of treatment ranging from ice, licorice, topical anesthetics agents, analgesics, and even milk can reduce the duration of cold sore. Zinc lozenges can also be used as they boost the immune systems. The current and widely used treatment of severe recurrent cold sore is oral or topical acyclovir. This drug is also used to treat genital herpes and herpes zoster. This agent is a guanosine analogue, which specifically inhibits herpes virus DNA polymerase. Acyclovir therapy neither eliminates latent virus nor prevents subsequent recurrences (12).

It is well known that cold sores manifest differently in duration and severity even in an individual. In this study, in order to reduce this confounding factor, we recruited cases that had similar profiles of recurrence and severity. This was achieved by preliminary completion of demographic characteristics and the history of their previous cold sores in terms of duration and severity, and cases that had recurrences with at least three-month intervals and sores durations ranging from seven to ten days were recruited.

Previous studies have shown that although acyclovir reduces the duration of healing and the intensity of pain associated with herpes labialis, it does not affect the progression stage of crust formation. This benefit was more significant when the patients started a five times a day oral treatment during the prodromal or erythematous stages than when initiated during papular or late macular stages. The mean duration was reduced from 7.9 to 5.8 days 13). However, regardless of the time of initiating treatment, no differences in the benefits between five times a day and topical application of 5% acyclovir were seen. The mean healing time were 4.3 and 4.8 days for medication and placebo, respectively (14). The results from the present study showed that the alcoholic solution of 0.5% shallomin produced a rapid clearance of cold sore in 30% of cases within six hours and all the sores were cleared within 24 hours when applied during the early stages. The selected concentration was made from our dose-finding pilot study. Further studies should be conducted to assess its effectiveness when applied during the late stages; however, these findings suggest that shallomin is a more potent antiviral agent that acyclovir.

In a recent study, a preliminary *in vivo* toxicological evaluation of escalating doses of shallomin was performed and demonstrated that shallomin was a relatively safe drug when administrated at its standard expected antimicrobial concentration by intraperitoneal route (8). This finding suggests that shallomin can be administered systemically for treatment of more severe forms of herpes infections. A preliminary, unpublished, clinical study by our group showed that shallomin is also effective in the treatment of HSV-2, a more common cause of genital herpes.

With regard to the clinical implication of these findings, herpes infections have been implicated as the causative agent in various diseases in different body systems such as neonatal herpes simplex, a rare but serious infection that is transmitted vertically from mother to newborn. It also produces unusual lesions on the skin during immunodeficiency such as herpes syncosis, a recurrent infection primarily affecting the hair follicles. Moreover, eczema herpeticum is a spreading form of herpes in the areas inflicted with chronic eczema.

The cumulative results from this study and those reported from other previous studies demonstrated that shallomin, at different concentrations, is a relatively safe and a potent chemotherapeutic agent of natural origin, having a wide range of activity against bacteria, fungi, and viruses, a quality that has not been reported previously for any of the known chemotherapeutic agents (6, 7, 9). Considering the safety profile of this agent, employment of this agent in conditions where HSV is implicated as the causative agent can be assessed in future studies and this agent may find its wider use in clinical settings. In conclusion, the findings from our study demonstrated that shallomin is a useful and effective treatment modality with more rapid response. Its usage is recommended in treatment of cold sores when applied within 24 hours of development.

## References

[1] Dickerson FB, Boronow JJ, Stallings C, Origoni AE, Cole S, Krivogorsky B (2004). Infection with herpes simplex virus type 1 is associated with cognitive deficits in bipolar disorder.. Biol Psychiatry..

[2] Itzhaki RF, Wozniak MA (2008). Herpes simplex virus type 1 in Alzheimer's disease: the enemy within.. J Alzheimers Dis..

[3] Leung DT, Sacks SL (2003). Current treatment options to prevent perinatal transmission of herpes simplex virus.. Expert Opin Pharmacother..

[4] Perfect MM, Bourne N, Ebel C, Rosenthal SL (2005). Use of complementary and alternative medicine for the treatment of genital herpes.. Herpes..

[5] Beauman JG (2005). Genital herpes: a review.. Am Fam Physician..

[6] Amin M, Kapadnis BP (2005). Heat stable antimicrobial activity of Allium ascalonicum against bacteria and fungi.. Indian J Exp Biol..

[7] Amin M, Kapadnis BP (2006). An investigation of antimicrobial properties of shallot..

[8] Amin M, Pipelzadeh MH, Mehdinejad M, Rashidi I (2012). An In Vivo Toxicological Study Upon Shallomin, the Active Antimicrobial Constitute of Persian Shallot (Allium hirtifolium, Boiss) Extract.. Jundishapur J Nat Pharm Prod..

[9] Amin M, Jahangirnezhad M, Rasaei N, Pipelzadeh MH, Rafiee M (2012). Evaluation of the effect of Persian shallot (Allium hirtifolium, boiss) aqueous extract on mouth bacterial count compared with chlorhexidine mouth rinse.. Afr J Microbiol Res..

[10] Taran M, Rezaeiian EA, Izaddoost M (2006). In vitro antitrichomonal activity of Allium hertifolium (Persian shallot) in comparison with metronidazole.. Iranian J Pub Health..

[11] Jellin JM, Batz F, Hitchens K (2002). Natural medicines comprehensive data base..

[12] Gnann JW, Jr, Barton NH, Whitley RJ (1983). Acyclovir: mechanism of action, pharmacokinetics, safety and clinical applications.. Pharmacotherapy..

[13] Spruance SL, Stewart JC, Rowe NH, McKeough MB, Wenerstrom G, Freeman DJ (1990). Treatment of recurrent herpes simplex labialis with oral acyclovir.. J Infect Dis..

[14] Spruance SL, Nett R, Marbury T, Wolff R, Johnson J, Spaulding T (2002). Acyclovir cream for treatment of herpes simplex labialis: results of two randomized, double-blind, vehicle-controlled, multicenter clinical trials.. Antimicrob Agents Chemother..

